# “Incidentally” discovered Von Hippel Lindau disease: an emerging clinical phenotype

**DOI:** 10.1093/oncolo/oyaf015

**Published:** 2025-03-10

**Authors:** Michael N Trinh, Lauren Bear, Brian V Nahed, Othon Iliopoulos

**Affiliations:** Division of Hematology-Oncology, Department of Medicine, Massachusetts General Hospital, Boston, MA 02114, United States; Department of Medicine, Harvard Medical School, Boston, MA 02115, United States; Division of Hematology-Oncology, Department of Medicine, Massachusetts General Hospital, Boston, MA 02114, United States; Center for Cancer Risk Assessment, Massachusetts General Hospital Cancer Center, Boston, MA 02114, United States; Department of Neurosurgery, Massachusetts General Hospital, Boston, MA 02114, United States; Division of Hematology-Oncology, Department of Medicine, Massachusetts General Hospital, Boston, MA 02114, United States; Department of Medicine, Harvard Medical School, Boston, MA 02115, United States; Center of Cancer Research, Massachusetts General Hospital Cancer Center, Charlestown, MA 02129, United States

**Keywords:** Von Hippel Lindau (VHL) disease, renal cancer, hemangioblastoma, germline pathogenic variants

## Abstract

Increasing accessibility to genetic screening for cancer risk can lead to earlier surveillance and prevention, but with this comes the caveat of incidental identification of germline pathogenic gene variants. Here, we report a single institution case series of 6 otherwise healthy individuals with “incidental” Von Hippel Lindau (VHL) disease. These patients were found to have pathogenic germline variants in the *VHL* gene, after undergoing genetic testing for other purposes (5 for familial breast cancer risk and 1 to determine ancestry) but no VHL disease-associated tumors. The penetrance and expressivity of such incidental variants are not currently known, and therefore, no surveillance guidelines exist. Nevertheless, the association of these variants historically with high disease penetrance compels us to currently recommend active surveillance of their carriers with annual imaging of the brain, spine, and abdomen.

## Introduction

Increased accessibility to hereditary cancer genetic testing has the potential for surveillance, early detection, and improved clinical outcomes. However, this comes with an increased chance of incidental findings for which there was a low pretest probability, leaving the clinician to decide on a plan of surveillance.^1,2^

**Figure 1.**
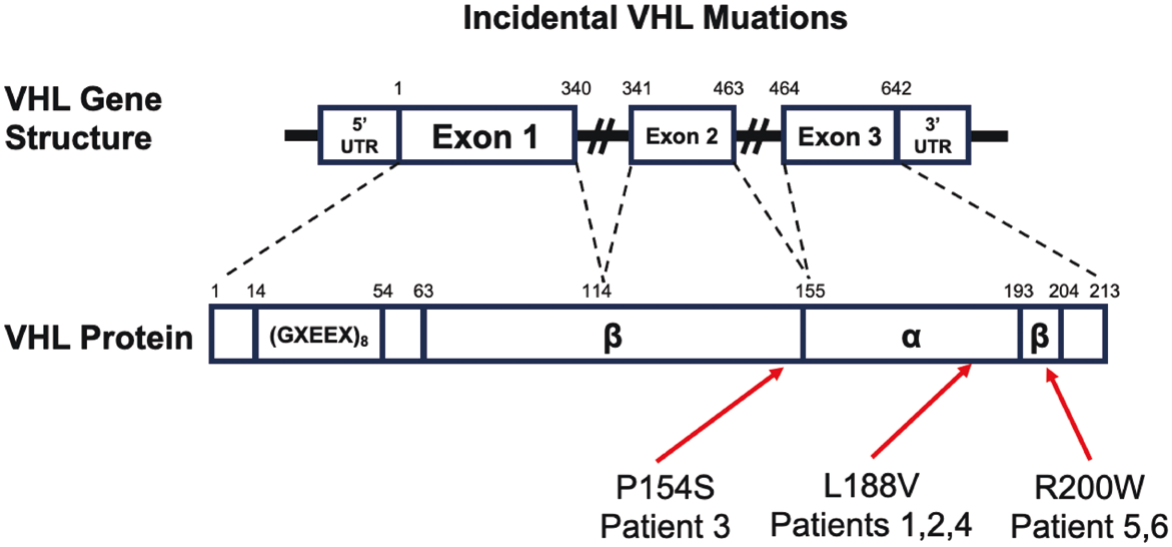
“Incidental” VHL disease mutations and their positions in the *VHL* gene and protein structure. Nucleotide and amino acid positions depicted in gene and protein, respectively. Pertinent protein features noted as well including GXEEX acidic repeat sequences.

Here, we describe 6 patients (Table 1) referred to us at a single institution (Familial Renal Cell Carcinoma & VHL Disease Program and Hemangioblastoma Center, at Mass General Cancer Center). Typically, at the time of referral to our centers, most patients with VHL would have been found to have a characteristic VHL lesion and/or a strong family history suggestive of VHL that prompted the referral. In the below cases, we describe a new clinical entity we call “Incidental Von Hippel Lindau Disease” in 6 otherwise healthy patients who pursued genetic screening with low a priori risk for VHL (5 individuals pursuing testing for hereditary breast cancer risk and 1 to determine ancestry). All 6 patients were found to carry incidental germline pathogenic variants in the *VHL* gene (Figure 1).

**Table 1.** Description of patients with “incidental” Von Hippel Lindau disease.

Patient No.	VHL mutation	Family history of cancer	Imaging findings: MRI of the abdomen, brain, and spine
1	c.562C>G, p.Leu188Val	Yes, breast	Negative
2	c.562C>G, p.Leu188Val	Yes, breast	Negative
3	c.460C>T, p.P154S	Unknown	Negative
4	c.562C>G, p.Leu188Val	Yes, breast	Spinal hemangioblastomas at the level of T6 (6mm) and T8 (9 mm)
5	c598C>T, p.R200W	Yes, breast, ovarian, colon	Negative
6	c598C>T, p.R200W	Yes, breast	Negative

Patient 1, a 40-year-old female and her 20-year-old daughter (Patient 2) presented to our clinic after testing positive for a pathogenic variant in *VHL* (c.562C>G, p.L188V via Multi-Cancer Panel, Invitae Genetics). The impetus for testing came after the mother and maternal aunt of patient 1 underwent genetic testing for early-onset breast cancer and tested positive for the aforementioned *VHL* pathogenic variant.

Patient 1 had notably previously undergone hysterectomy and bilateral salpingo-oophrectomy for endometriosis and her cervix was found to have a high-grade squamous intraepithelial lesion. Her past medical history was only notable for peripheral neuropathy and atrophic optic neuritis. Her physical exam revealed a mild peripheral neuropathy of unknown significance characterized by hypesthesia and pain in the left leg and weakness in the contralateral arm. Assessment for VHL-related lesions, including MRI of the brain, spine, and abdomen, was negative, except for punctate pancreatic intraductal mucinous neoplasms and her complete blood count and metabolic panel were normal.

Patient 2 had a noncontributory medical history other than ADHD and minor sports injuries and a physical exam was normal. Laboratory studies and MRI of the brain, spine, and abdomen were negative for VHL-related lesions. Both patients we referred to ophthalmology for retinal exam and recommended yearly MRIs of the brain, spine, and abdomen, as well as laboratory studies.

Patient 3, a 43-year-old female with no relevant past medical history, presented with a germline *VHL* likely pathogenic variant (c.460C>T, p.P154S via multigene panel, Ambry Genetics). She was adopted without information regarding her biological relatives and originally sought ancestry testing through 23andMe, revealing a germline *BRCA1* pathogenic variant. Retesting through Ambry identified and confirmed the *VHL* and *BRCA1* (c.4035delA, p.E1346Kfs*20) variants, respectively. She underwent prophylactic bilateral mastectomies and bilateral salpingo-oophrectomy. A physical exam was normal. Laboratory studies and MRI of the brain, spine, and abdomen were negative for VHL-related lesions. We referred her to ophthalmology for retinal exam and recommended annual MRIs of the brain, spine, and abdomen, as well as laboratory studies. Her 2 children were also recommended to be tested for these variants.

Patient 4, a 37-year-old female, presented to our clinic after testing positive for a *VHL* pathogenic variant (c.562C>G, p.L188V via Multi-Cancer Panel, Invitae Genetics). The impetus for her testing was her extensive maternal and paternal family history of early onset breast cancer and the previous discovery of the same *VHL* pathogenic variant in her sister. Her medical history is notable for latent tuberculosis infection and exercise-induced asthma. A physical exam and laboratory work were unremarkable and MRI of her brain, spine, and abdomen were unremarkable except for small, asymptomatic spinal hemangioblastomas at the level of T6 (6 mm) and T8 (9 mm). We recommended yearly imaging, retinal exam, and laboratory studies.

Patient 5, a 42-year-old female with a medical history of systemic discoid lupus erythematosus, rheumatoid arthritis, and chronic sciatic pain due to spinal synovial cysts treated with foraminotomies, laminectomies, and discectomy at L3-4 and at L4-5, presented with 2 heterozygous pathogenic variants (Multi-Cancer Panel, Invitae Genetics): (1) a substitution in *VHL* (c.598C>T, p.R200W) and (2) a splice site pathogenic variant in *BLM* (c.2192+1_2193+9del). This was pursued in light of a family history of early onset breast, ovarian, and colon cancer in a paternal aunt. The single *BLM* variant reveals that she is a carrier for Bloom syndrome (autosomal recessive condition), therefore is unlikely to affect her health based upon current evidence. The *VHL* pathogenic variant she carries is classically associated with autosomal recessive Chuvash Erythrocytosis,^3^ and its association with VHL disease remains somewhat unclear. Her physical exam and laboratory work were unremarkable, and MRI of her brain, spine, and abdomen did not detect any VHL-related lesions. We recommended yearly imaging, retinal exam, and laboratory studies.

Patient 6, a 59-year-old female with a history of endometriosis and possible fibroids, for which she underwent total abdominal hysterectomy and bilateral salpingo-oophrectomy years prior to presentation, presented with (1) a substitution in *VHL* (c.598C>T, p.R200W, aforementioned Chuvash variant) and (2) a likely pathogenic variant in *FH* (c1431_1433dup, pLys477dup) after seeking cancer genetic testing given a second-degree relative had early-onset breast cancer (Multi-Cancer Panel, Invitae Genetics). Her physical exam and laboratory work were unremarkable and MRI of her brain, spine, and abdomen did not detect any VHL-related lesions. Germline *FH* pathogenic variants are linked to the development of Hereditary Leiomyomatosis and Renal Cell Carcinoma (HLRCC) disease.^4^ However, this specific variant has not been unequivocally linked to HLRCC risk,^5^ although we need more definitive and confirmatory data to definitely conclude the risk for HLRCC for this variant. We recommended yearly imaging, retinal exam, and laboratory studies.

## Discussion

Historically, patients referred for genetic counseling and germline *VHL* testing were at risk of a known familial VHL variant, or patients with a personal or family history raising suspicion for VHL disease (eg, early-onset ccRCC [≤46-years-old], hemangioblastoma, pheochromocytoma, or 2+ VHL-associated lesions). This population was characterized by a referral bias based on presence of tumor(s) in the proband or their families. The prevalence (1:36 000), high penetrance (almost 97%), and expressivity of VHL disease were tabulated based on this referral bias.^6^

Here, we describe 6 patients incidentally discovered to carry germline pathogenic *VHL* mutations, presenting to our institution over the last 2 years. Standard VHL imaging revealed no evidence of VHL tumors at presentation. For such unsuspected *VHL* germline variants without clinical stigmata of VHL disease, we coin the term “incidental VHL disease.” It is unclear whether these patients will develop VHL-related tumors during their lifetime, however given the historical penetrance and associated morbidity and mortality of VHL lesions, we opted to apply the standard VHL patient surveillance protocol.

Currently, the expanding practice of germline testing via multigene panels that include hereditary cancer risk genes across multiple cancer types provides insights into the genomes of completely asymptomatic patients. The result is the identification of germline variants in unexpected genes, linked to unsuspected hereditary risks for cancer. This is not a VHL-specific phenomenon and encompasses many cancer-predisposing genes.^2,7^ Penetrance, namely the percentage of individuals harboring the pathogenic variant who express any phenotype of the disease, may be influenced by many factors, including gender, age, genetic (specific germline variant, genetic background inherited from the parents), and environmental variables.^8-10^ These “incidental” findings in patients without disease-specific lesions are likely to challenge our established view of the penetrance and expressivity of inherited cancer predisposition syndromes. Moreover, established guidelines on how to clinically follow these patients are lacking. We recommend referral to specialized high-risk cancer clinics for surveillance recommendations.

Most of the patients presented here carry classic *VHL* pathogenic variants. It is noteworthy, though, that 2 of these patients carry the *VHL* c.598C>T, p.R200W pathogenic variant, which is linked to Chuvash polycythemia, pulmonary hypertension, and thrombotic episodes when present in the homozygous/compound heterozygous state.^3^ Classic VHL-associated tumors have so far not been reported in families with heterozygous Chuvash variant, indicating that this germline variant may lead to VHL disease, albeit with reduced penetrance.^10^ Anecdotally, we follow one family with this Chuvash variant at our center, in which the affected members developed renal cell carcinoma and metastatic pancreatic neuroendocrine tumor (O.I. observation).

In conclusion, this case-series highlights a growing phenomenon in cancer genetic counseling and testing: identification of incidental *VHL* variants. Long-term surveillance of these patients is required in order to understand the penetrance of incidentally discovered pathogenic variants not only for VHL but also for other hereditary cancer predisposition syndromes.

## Data Availability

Upon request from Dr. Iliopoulos.
